# Mitochondrial Dysfunction Contributes to Aging-Related Atrial Fibrillation

**DOI:** 10.1155/2021/5530293

**Published:** 2021-04-28

**Authors:** Chuanbin Liu, Jing Bai, Qing Dan, Xue Yang, Kun Lin, Zihao Fu, Xu Lu, Xiaoye Xie, Jianwei Liu, Li Fan, Yang Li

**Affiliations:** ^1^Medical School of Chinese PLA, Beijing, China; ^2^National Clinical Research Center for Geriatric Disease, Chinese PLA General Hospital, Beijing, China; ^3^Department of Cardiology, The Sixth Medical Center, Chinese PLA General Hospital, Beijing, China; ^4^The Second Medical Center, Chinese PLA General Hospital, Beijing, China

## Abstract

The incidence of atrial fibrillation (AF) increases with age, and telomere length gradually shortens with age. However, whether telomere length is related to AF is still inconclusive, and the exact mechanism by which aging causes the increased incidence of AF is still unclear. We hypothesize that telomere length is correlated with aging-related AF and that mitochondrial dysfunction plays a role in this. This research recruited 96 elderly male patients with AF who were admitted to the Second Medical Center of Chinese PLA General Hospital from April to October 2018. After matching by age and gender, 96 non-AF elderly male patients who were admitted to the hospital for physical examination during the same period were selected as controls. Anthropometric, clinical, and laboratory analyses were performed on all subjects. The mitochondrial membrane potential (MMP) of peripheral blood leukocytes was detected as the indicator of mitochondrial function. Compared with the control group, the leukocyte telomere length (LTL) was significantly shorter (*P* < 0.001), and the level of PGC-1*α* in serum was significantly lower in AF patients. Additionally, in subjects without any other diseases, the AF patients had lower MMP when compared with the control. Multivariate logistic regression confirmed that LTL (OR 0.365; 95% CI 0.235-0.568; *P* < 0.001) and serum PGC-1*α* (OR 0.993; 95% CI 0.988-0.997; *P* = 0.002) were inversely associated with the presence of AF. In addition, ROC analysis indicated the potential diagnostic value of LTL and serum PGC-1*α* with AUC values of 0.734 and 0.633, respectively. This research concludes that LTL and serum PGC-1*α* are inversely correlated with the occurrence of aging-related AF and that mitochondrial dysfunction plays a role in this.

## 1. Introduction

Atrial fibrillation (AF) is the most common cardiac arrhythmia and contributes to a high prevalence of mortality and morbidity [1]. Studies have shown that the prevalence of AF increases with advancing age, reaching 5% between 60 and 70 years old and as high as 8% at over 80 years old [2]. Furthermore, the incidence of AF in males is higher than in females [3].

The shortening of telomere length has been found to be common with age in the majority of tissues and cells; thus, it is often used as a biomarker of aging [4]. Research efforts have argued that leukocyte telomere length (LTL) shortening is related to a variety of cardiovascular diseases, including atherosclerosis, left ventricular hypertrophy, and heart failure, but the relevance to AF is still controversial [5–7]. In the Cardiovascular Health Study, researchers found no relationship between mean telomere length and AF in human atrial tissue [8]. However, Carlquist et al. found that shortened LTL was related to the presence of paroxysmal AF among cardiovascular patients [9]. In addition, recent studies have argued that shortened LTL is associated with the recurrence of AF and is an independent risk factor in humans [10].

The mechanisms of AF remain incompletely understood. Mitochondria play an important role in oxidative stress, calcium homeostasis, and energy metabolism. Studies have shown that mitochondrial dysfunction can cause insufficient ATP production and excessive reactive oxygen species (ROS), which damages the homeostasis of Ca^2+^ in myocardial cells and the excitability of membranes, in turn leading to AF [11, 12]. Peroxisome proliferator-activated receptor *γ* coactivator-1 (PGC-1) is an important nuclear transcription coactivator that contains PGC-1*α*, PGC-1*β*, and PGC-1-related coactivator (PRC) [13]. Accumulating evidence has argued that PGC-1*α* is a key molecule of mitochondrial function because it participates in the regulation of mitochondrial biogenesis and energy metabolism and is closely related to oxidative stress and inflammation [14, 15]. It plays an important role in the occurrence and development of atherosclerosis, coronary heart disease, heart failure, and other cardiovascular diseases [15, 16]. Some researchers have put forward the concept of a “telomere-p53-PGC axis”: that is, that the shortening of telomere will activate p53 expression, thereby inhibiting PGC-1 and causing mitochondrial dysfunction and a series of reactions such as oxidative stress and intracellular Ca^2+^ overload, eventually inducing AF [11, 12, 17, 18].

It is not clearly known whether telomere shortening is associated with aging-related AF and whether mitochondrial dysfunction is involved in this process. Therefore, we measured the LTL, telomere-associated molecules, and mitochondrial membrane potential (MMP) of leukocytes to ascertain if they are correlated with aging-related AF and if they could be used as novel biomarkers for it.

## 2. Materials and Methods

### 2.1. Ethics Approval of the Research Protocol

The study protocol was approved by the Human Ethics Review Committee of Chinese PLA General Hospital, and a signed consent form was obtained from each subject.

### 2.2. Participants

**Figure 1:**
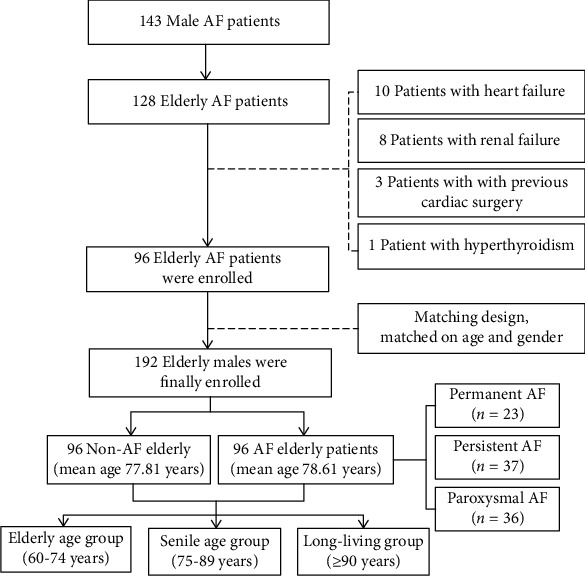
Study frame diagram. AF: atrial fibrillation.

A total of 143 male patients with AF were admitted to the Second Medical Center of Chinese PLA General Hospital from April to October 2018. The inclusion criteria were (i) patients aged 60 years or older and diagnosed as having AF by electrocardiogram or 24 h dynamic electrocardiogram according to the guidelines established by the European Society of Cardiology in 2010 [19] and (ii) patients with a complete clinical data record. According to the inclusion criteria, 118 patients were enrolled in the study. The exclusion criteria were valvular heart disease, acute coronary syndrome, dilated or hypertrophic cardiomyopathy, congenital heart disease, previous cardiac surgery, heart failure (including heart failure with preserved ejection fraction and heart failure with reduced ejection fraction), hyperthyroidism, inflammatory diseases, systemic disease, and moderate-to-severe renal dysfunction (estimated glomerular filtration rate (eGFR) < 60 mL/min/1.73 m^2^). Considering the effect of diseases on AF, 10 patients with heart failure, 8 patients with renal dysfunction, and 1 patient with hyperthyroidism were excluded. After excluding another 3 patients with previous cardiac surgery, 96 participants with AF were enrolled in the study as an AF group. At the same time, we selected 96 non-AF elderly males who were admitted to the hospital for physical examination during the same period as controls after matching for age and gender. LTL was associated with AF with a hazard ratio (HR) of 3.17 in a previous study [10]. According to the sample size calculation formula for a paired case-control study, the sample size required was calculated to be 85 patients by using PASS 11 software (proportioning; tests for two correlated proportions in a matched case-control design). Consequently, having 96 subjects in each group met the sample size requirements; therefore, 192 elderly males were selected as the study cohort. Among them, 96 AF patients (mean age 77.81 years, range 61-97 years) were in the AF group, and 96 elderly males (mean age 78.61 years, range 60-103 years) were in the control group (Figure 1). The subjects were divided into the elderly age group (60-74 years), the senile age group (75-89 years), and the long-living group (≥90 years) [20]. AF patients were then divided into the paroxysmal AF group (*n* = 36), the persistent AF group (*n* = 37), and the permanent AF group (*n* = 23).

### 2.3. Clinical Data Collection

Patients' demographic characteristics, lifestyle information, and medication use were obtained by reviewing their medical records. The age value was the age recorded at admission. The definition of “smoking” was having smoked more than 1 cigarette per day for over 1 year. Patients' body mass index (BMI) was also calculated, and blood pressure was measured using the right arms of seated participants by an automated blood pressure monitor (J710, Omron Corporation, Kyoto, Japan) in the morning. Transthoracic echocardiography was performed by experienced echocardiologists on all subjects to evaluate the characteristics of their left atrial diameter (LAD) and left ventricular ejection fraction (LVEF).

### 2.4. Biochemical Index Determination

Blood samples were obtained with anticoagulation between 6:00 and 7:00 a.m. after patients fasted overnight. Samples were stored at 4°C for less than 1 hour. Some samples were used for testing, and others were centrifuged to obtain white blood cells and plasma and frozen at -80°C. Concentrations of fasting blood glucose (FBG), uric acid (UA), blood lipid, blood urea nitrogen (BUN), and C-reactive protein (CRP) were measured by enzymatic assays (Roche Diagnostics, Mannheim, Germany). The concentration of creatinine was determined by an enzymatic assay (Roche Diagnostics) on an autoanalyzer (7600, Hitachi, Tokyo, Japan). The glycated hemoglobin (GHb) level was tested using high-performance liquid chromatography.

### 2.5. Leukocyte Isolation and Mitochondrial Membrane Potential Detection

The blood samples were diluted with an equal volume of buffer. Then, an appropriate amount of Ficoll-Hypaque (Solarbio, Beijing, China) was added to a tube, and the diluted blood was also carefully added to it. 800 g was centrifuged for 20 minutes at room temperature to isolate leukocytes. The cells in the middle layer were carefully pipetted into a new centrifuge tube and diluted with PBS. Then, 250 g was centrifuged for 10 minutes. The pellet was treated with PBS, and 250 g was centrifuged for 10 minutes, and peripheral blood mononuclear cells were achieved [21]. After being washed and resuspended in PBS, cells were treated with 500X MitoTell™ Orange (AAT Bioquest, Sunnyvale, USA) for 15-30 minutes at 37°C in the dark [22]. After washing, the fluorescence was measured using a flow cytometer at an emission of 590 nm and 540 nm. The data was analyzed using the software FlowJo V10.

### 2.6. Measurement of Telomere Length

The leukocytes were obtained from blood samples by centrifugation and stored at -80°C until analysis. Telomere length in genomic DNA was extracted directly from peripheral blood leukocytes according to the instructions of the DNA extraction kit (TIANGEN Biotech Corporation, China) and was measured by applying a quantitative real-time PCR method (GenePool Biotech Corporation, China). Telomere length was measured according to the ratio of the telomere repeat copy number (*T*) to the single-copy gene copy number (*S*) in each given sample. The relative LTL was calculated as the ratio of telomere repeats to single-copy gene copies (*T*/*S* ratio) [10]. DNA samples were amplified in 10 *μ*L PCR reactions with StepOnePlus Real Time PCR System (Applied Biosystems, Foster City, CA, USA). The primers used for the telomere repeat and the single-copy gene copy number amplification were as follows: telomere forward—ACACTAAGGTTTGGGTTTGGGTTTGGGTTTGGGTTAGTGT, telomere reverse—TGTTAGGTATCCCTATCCCTATCCCTATCCCTATCCCTAACA; single-copy gene forward—CTTCATCCACGTTCACCTTG, single-copy gene reverse—GAGGAGAAGTCTGCCGTT [10]. Both PCRs were activated in a final volume of 10 *μ*L that contained SYBR Green Master Mix none-ROX (2x) (TaKaRa, Shiga, Japan), 3.12 ng of DNA template, and 0.5 nM of telomere primers or 0.5 nM of single-copy gene primers. The thermal cycling profile for both telomere and single-copy gene primers started with 95°C incubation for 10 minutes, followed by 40 cycles of 15 seconds at 95°C and 1 minute at 54°C. All amplification specificity was regulated by employing melting curve analysis. In each sample, the quantities of telomere repeats and single-copy genes were normalized to a reference DNA. The same reference DNA sample (from a single individual) was included in each measurement to control interassay variability [9]. All measurements were performed blinded with respect to clinical data.

### 2.7. RNA Isolation and mRNA Expression Analysis

The leukocytes were obtained from blood samples by centrifugation and stored at -80°C until analysis. Total RNA was extracted from leukocytes by RNAprep Pure Blood Kit (TIANGEN Biotech Corporation, China) according to the standard protocol. Absorption spectrophotometry, using NanoDrop-1000 (Thermo Fisher Scientific, Yokohama, Japan), was used to determine RNA concentrations and purity (260/280 ratio above 1.8 was used). RNA (1 *μ*g per sample) was reverse transcribed using the High-Capacity cDNA Reverse Transcription Kit (Applied Biosystems, CA, USA) in a total reaction volume of 40 *μ*L. For a quantitative estimate of p53 and PGC-1*α* mRNA levels, the StepOnePlus Real Time PCR System was used. The primers were as follows: p53 forward—CCATCCTCACCATCATCACACT, p53 reverse—GCACAAACACGCACCTCAAA; PGC-1*α* forward—TGACGACGAAGCAGACAAGAC, PGC-1*α* reverse—GAACAAGAAGGAGACACATTGAACA; Actin forward—ACTTAGTTGCGTTACACCCTT, and Actin reverse—GTCACCTTCACCGTTCCA. The relative expression level of p53 or PGC-1*α* mRNA was determined by comparison, with the housekeeping gene Actin serving as an internal standard. Relative mRNA levels were calculated via the 2^−*ΔΔ*CT^ method using StepOne software (Applied Biosystems) [23]. We averaged the fold changes from three wells for each sample (in triplicate) and used this average value for statistical analyses.

### 2.8. ELISA Measurement of Serum PGC-1*α*

An enzyme-linked immunosorbent assay kit (Jianglai Biotech Corporation, China) was utilized to evaluate serum PGC-1*α* concentrations. An anti-human PGC-1*α* monoclonal coating antibody adsorbed onto microwells and a lyophilized HRP-conjugated monoclonal anti-human PGC-1*α* were incubated with 100 *μ*L of diluted (1 : 20) sample serum at room temperature for 3 hours on a microplate shaker at 100 rpm. Following three washes, the unbound enzyme conjugate anti-human sPGC-1*α* was removed and 100 *μ*L of TMB (tetrametylbenzidine) substrate solution reactive with HRP was added to the wells. After incubation at room temperature for 10 minutes, the reaction was terminated by the addition of acid, and absorbance was measured at 450 nm. A standard curve was prepared from seven human PGC-1*α* standard dilutions, and human PGC-1*α* sample concentration was determined [24]. All measurements were performed blinded with respect to clinical data.

### 2.9. Definition of Variables

Paroxysmal AF was defined as AF terminating spontaneously within 7 days, especially within 48 hours. Persistent AF was defined as AF that lasted longer than 7 days (regardless of whether it terminated spontaneously or by cardioversion). Permanent AF was defined as AF that fails to terminate using cardioversion or is terminated but relapses within 24 hours [25, 26]. Hypertension was defined as systolic blood pressure (SBP) of ≥140 mmHg, diastolic blood pressure (DBP) of ≥90 mmHg, or taking antihypertensive medication.

### 2.10. Statistical Analysis

Statistical analyses were performed with the SPSS statistical package, version 20.0 (SPSS Inc., Chicago, USA). The continuous variables were exhibited as means ± standard errors, and categorical variables were expressed as percentages. The Student *t*-test and the chi-square test were utilized to determine the parameter differences between the AF patients and controls. Simple and multiple logistic regression analyses were performed to determine the correlation of LTL with the presence of AF. Chi-square tests, one-way ANOVA, or Kruskal-Wallis tests were utilized to determine the parameter differences between different age and AF subgroups. Pearson correlation analysis was used to analyze the correlation of LTL with age. The correlation between LTL and other parameters was analyzed using simple linear regression analysis. Then, a multiple stepwise linear regression analysis was used to determine the contribution of various factors to LTL. Receiver operating characteristic (ROC) curves were constructed to evaluate the specificity and sensitivity of predicting AF using LTL as well as the serum PGC-1*α* and CRP values, and the area under curve (AUC) was calculated. A *P* value of less than 0.05 was statistically significant.

## 3. Results

### 3.1. Baseline Clinical Characteristics

A total of 192 subjects were enrolled in this study, and their characteristics are summarized in Table 1. The AF patients showed higher UA and LAD compared with the controls. There were no significant differences in other characteristics between the two groups.

### 3.2. AF Patients Had Shorter LTL

**Figure 2:**
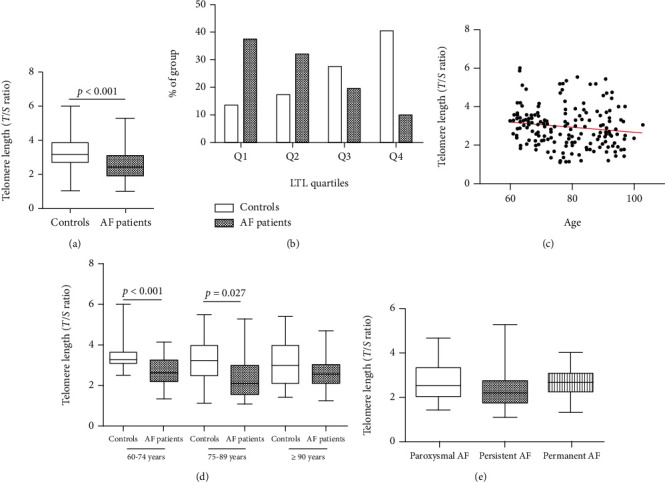
LTL in AF patients and the controls. (a) AF patients showed significantly shorter LTL compared with controls; (b) quartile distribution of LTL; (c) LTL in elderly males was significantly negatively correlated with age; (d) LTL was significantly shorter in AF patients in the elderly age group and senile age group; (e) there were no significant differences of LTL among the paroxysmal AF, persistent AF, and permanent AF groups. LTL: leukocyte telomere length; AF: atrial fibrillation; *Q*: quartile.

AF patients showed significantly shorter LTL compared with controls (2.54 ± 0.85 vs. 3.33 ± 1.01, *P* < 0.001) (Figure 2(a)). All subjects are separated into quartiles (*Q*) according to the LTL. The distribution of AF patients and controls from Q1 to Q4 is shown in Figure 2(b). As shown, the LTL of AF patients is mostly located in Q1 (37.50%) and Q2 (32.29%), while the controls' LTL is mostly located in Q3 (28.13%) and Q4 (40.63%). This indicates an inverse relationship between LTL and AF prevalence.

A Pearson correlation analysis showed that LTL in elderly males was significantly negatively correlated with age (*r* = −0.151, *P* = 0.037) (Figure 2(c)). In the control group, LTL was significantly shortened with age (*r* = −0.202, *P* = 0.048). However, in AF patients, LTL was also negatively correlated with age, but with no statistical difference (*r* = −0.089, *P* = 0.389).

Then, the elderly males were divided into three groups according to the WHO's guidelines [20]. The LTL in AF patients was significantly shorter in the elderly age group (60-74 years) (*P* < 0.001) and the senile age group (75-89 years) (*P* = 0.027) than in the controls. It was also shorter in AF patients in the long-living group (≥90 years), but without being statistically significant (Figure 2(d)). In the AF patient group and the control group, analysis of variance on the LTL of the three age groups showed no statistical difference. In the AF subgroups, there were no significant differences in LTL among the paroxysmal AF, persistent AF, and permanent AF groups (Figure 2(e)).

### 3.3. Telomere-Associated Molecules

**Figure 3:**
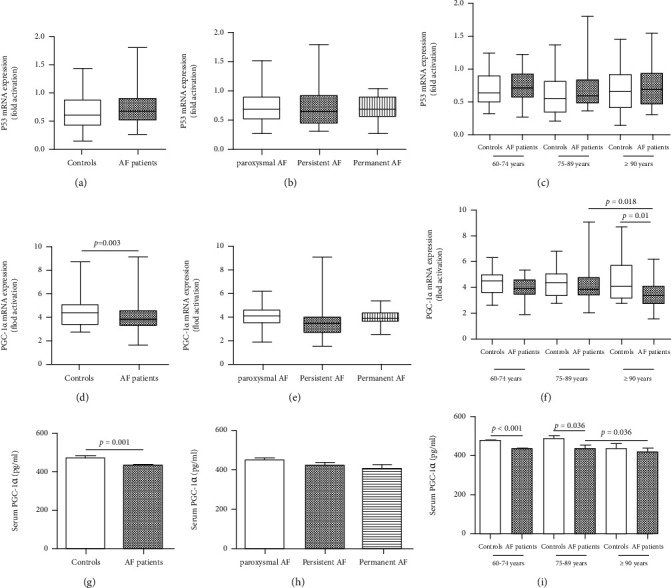
Telomere-associated molecules in AF patients. (a) p53 mRNA in AF patients was higher than controls and without statistical differences; (b, c) no significant differences of p53 mRNA among different types of AF and different age groups; (d) the expression of PGC-1*α* mRNA in leukocytes was significantly reduced in AF patients; (e) no significant differences of PGC-1*α* mRNA among different types of AF; (f) the expression of PGC-1*α* mRNA was significantly lower in AF patients in the long-living age group; (g) the serum PGC-1*α* concentration was significantly reduced in AF patients; (h) no significant differences of serum PGC-1*α* concentration among different types of AF; (i) the serum PGC-1*α* concentration was significantly reduced in AF patients in the elderly age group and senile age group. AF: atrial fibrillation.

The expression of p53 mRNA in leukocytes in AF patients was higher than that in the controls, but there was no statistical difference (Figure 3(a)). Compared with the controls, the expression of PGC-1*α* mRNA in leukocytes was significantly lower (4.38 ± 1.17*vs.*3.87 ± 1.15, *P* = 0.003) (Figure 3(d)), as was the serum PGC-1*α* concentration (470.41 ± 84.13*vs.*428.56 ± 86.07, *P* = 0.001) (Figure 3(g)). Interestingly, in AF patients, the p53 mRNA, PGC-1*α* mRNA, and serum PGC-1*α* levels had no significant differences among the paroxysmal AF, persistent AF, and permanent AF (Figures 3(b), 3(e), and 3(h)). Via subgroup analysis based on age, we found that the expression of PGC-1*α* mRNA was significantly lower in the long-living age group, and the serum PGC-1*α* concentration was significantly reduced in the elderly age group and the senile age groups in AF patients (Figures 3(f) and 3(i)). However, no significant differences were found in p53 mRNA among the different age groups (Figure 3(c)).

### 3.4. Subgroup Analysis for the Subjects without Other Diseases

**Figure 4:**
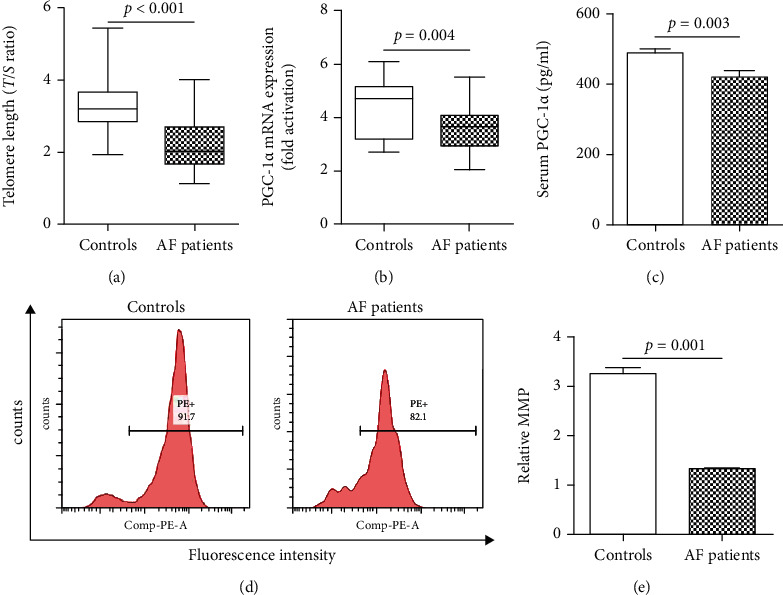
LTL, PGC-1*α* expression, and MMP in subgroup of patients without other diseases. (a) AF patients showed significantly shorter LTL compared with controls; (b) the expression of PGC-1*α* mRNA in leukocytes was significantly reduced in AF patients; (c) the serum PGC-1*α* concentration was significantly reduced in AF patients; (d) representative pictures of MMP detected by flow cytometry; (e) the MMP of AF patients was significantly decreased when compared with controls. AF: atrial fibrillation; MMP: mitochondrial membrane potential.

In order to reduce the impact of other diseases that may affect telomere length on the results of the study, we conducted a subgroup analysis of all nonsmoking subjects without comorbidities and measured the MMP of their peripheral blood leukocytes. Among these subjects, there was no significant difference in baseline data except CRP between the AF group and the control group (Table 2). The AF patients showed significantly shorter LTL and lower expression of PGC-1*α* mRNA and serum PGC-1*α* (Figures 4(a)–4(c)). In addition, the MMP of the AF patients was significantly decreased when compared with that of the controls, which indicates that AF patients have poor mitochondrial function (Figures 4(d) and 4(e)).

### 3.5. The Correlation of LTL with the Presence of AF

**Figure 5:**
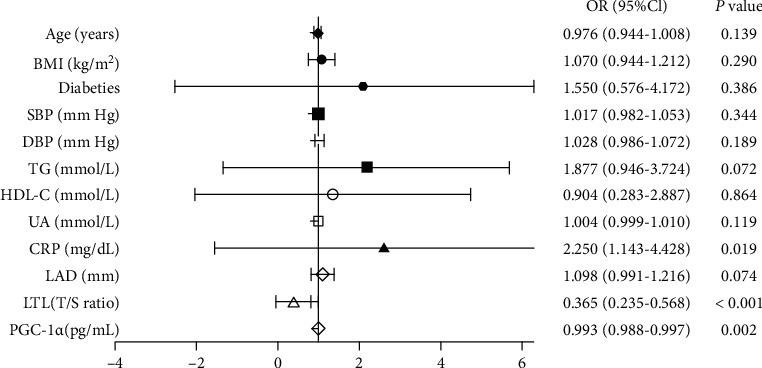
Multiple logistic regression analysis for the presence of AF. BMI: body mass index; SBP: systolic blood pressure; DBP: diastolic blood pressure; TG: triglyceride; HDL-C: high-density lipoprotein cholesterol; UA: uric acid; CRP: C-reactive protein; LAD: left atrial diameter; LTL: leukocyte telomere length.

Simple logistic regression analysis demonstrated that UA, CRP, LAD, LTL, and serum PGC-1*α* showed a trend toward an association with the presence of AF (Table 3). All of these parameters were then entered into a multiple logistic regression model, and the LTL (OR 0.404, 95% CI 0.278-0.587; *P* <0.001), CRP (OR 1.971, 95% CI 1.023-3.799; *P* = 0.043), and serum PGC-1*α* (OR 0.994, 95% CI 0.989-0.998; *P* = 0.003) remained to be significantly associated with the presence of AF (Table 3). Drawing from previous studies, variables that were considered clinically relevant such as age, BMI, diabetes, SBP, DBP, TG, and HDL-C were entered into a multivariate logistic regression model. LTL (OR 0.365, 95% CI 0.235-0.568; *P* < 0.001), CRP (OR 2.250, 95% CI 1.143-4.428; *P* = 0.019), and serum PGC-1*α* (OR 0.993, 95% CI 0.988-0.997; *P* = 0.002) were still significantly associated with the presence of AF (Figure 5).

In the subgroup of subjects without other diseases, we incorporated age, CRP, LTL, leukocyte PGC-1*α* mRNA expression, and serum PGC-1*α* into the multivariate logistic regression model and also found that LTL and serum PGC-1*α* were significantly associated with the presence of AF (Table 4).

### 3.6. The Correlation of LTL with Other Parameters

Simple linear regression analyses showed that LTL was negatively correlated with age (*r* = −0.151, *P* = 0.037), CRP (*r* = −0.200, *P* = 0.005), and LAD (*r* = −0.196, *P* = 0.006) and positively correlated with PGC-1*α* mRNA (*r* = 0.168, *P* = 0.020) and serum PGC-1*α* (*r* = 0.176, *P* = 0.014). Multiple stepwise regression analysis showed that CRP (*β* = −0.167, *P* = 0.017) and LAD (*β* = −0.165, *P* = 0.018) remain to be inversely associated with LTL (Table 5).

### 3.7. LTL and Serum PGC-1*α* Has a Potential Predictive Role in Elderly AF

**Figure 6:**
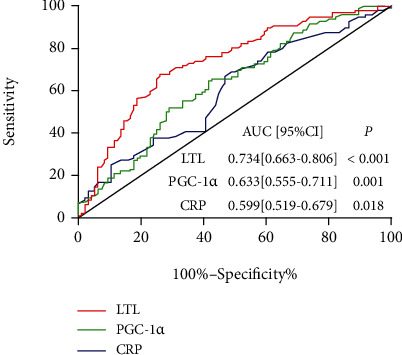
ROC curves of LTL, PGC-1*α*, and CRP to predict AF. ROC analysis was performed to determine the sensitivity and specificity of the value. ROC: receiver operator characteristic; AUC: area under the curve; CI: confidence interval; LTL: leukocyte telomere length; CRP: C-reactive protein.

The corrected multivariate logistic regression model has argued that LTL, CRP, and serum PGC-1*α* are significantly related to the presence of AF in the elderly. ROC analysis was used to determine if LTL, CRP, and serum PGC-1*α* could predict AF. As shown in Figure 6, the AUC was 0.734 (95% CI: 0.663-0.806, *P* < 0.001) for LTL in the prediction of AF. The optimum cutoff value of LTL on the ROC curve was 2.830 with a sensitivity of 67.7% and a specificity of 74.0%. Serum PGC-1*α* showed a potential predictive value of AF with an AUC of 0.633 (95% CI: 0.555-0.711; *P* = 0.001), and the optimum cutoff value was 463.5 with a sensitivity of 65.6% and a specificity of 57.3%. Further, the AUC for CRP in the prediction of AF was 0.599 (95% CI: 0.519-0.679; *P* = 0.018), and the optimum cutoff value was 0.6150 with a sensitivity of 68.8% and a specificity of 51.0% (Figure 6).

## 4. Discussion

This study found that LTL and serum PGC-1*α* are inversely correlated with the occurrence of aging-related AF and that the MMP of AF patients was significantly decreased, indicating that mitochondrial dysfunction plays a role in this. In addition, ROC analysis revealed the potential diagnostic value of LTL and serum PGC-1*α* for AF patients, indicating that LTL and serum PGC-1*α* could possibly be novel predictive biomarkers for the occurrence or outcome of aging-related AF.

Telomere shortening has been suggested to be susceptible to age-related cardiovascular diseases, including atherosclerosis and heart failure [5, 7]. Many studies have shown that LTL predicts cardiovascular disease and all-cause mortality [27–30]. However, it is not clear whether telomere shortening is related to the occurrence of AF. In the current study, we found that LTL was significantly shorter in elderly male AF patients compared with the controls, and the multivariate logistic regression analysis confirmed that LTL was significantly related to AF. Our finding is consistent with an analysis of the Intermountain Heart Collaborative Study investigators in a 63% male cohort with a mean age of 62.9 ± 13.47 years [9]. However, Siland et al. [31] found that shorter LTL was not independently associated with incident AF in a community-based, 50% male cohort with a mean age of 49 ± 13 years (range between 29 and 74 years). Roberts *et al.* [8] found no evidence of an association between LTL and incident AF in a cohort with the mean age of 72.2 years that was 41.3% male at baseline. In our opinion, this difference in results may come from the choice of subjects, as the subjects were not elderly or had a lower ratio of males. In the subgroup analysis of age, we found that the LTL of patients with AF in the elderly age group and the senile age group was significantly shorter when compared with that of the controls, while there was no statistical difference in the long-living group, indicating that the rate of telomere shortening is different at different ages and that telomere may shorten faster in the early stages of aging [32].

The mechanisms of aging-related AF remain incompletely understood, but atrial electrical and structural remodeling and disturbed calcium homeostasis may be involved [33, 34]. Mitochondrial function is the core of energy metabolism, which is involved in oxidative stress, the regulation of intracellular calcium homeostasis, and intracellular signal transduction. Studies have shown that mitochondrial dysfunction causes energy metabolism disorders and decreased membrane potential, leading to myocardial abnormal local electrical activity [35]. In addition, mitochondrial dysfunction can cause oxidative stress and calcium overload, thereby promoting the occurrence of AF [36]. Studies have argued that telomere shortening consequently contributes to mitochondrial dysfunction, and the “telomere-p53-PGC axis” plays an important role in this [37]. Thus, telomere shortening will activate p53, thereby inhibiting PGC-1 and inducing mitochondrial dysfunction as well as a series of reactions such as oxidative stress and Ca^2+^ overload [11, 17]. Researchers had also found that the telomere of left ventricular cardiomyocytes of TERC^−/−^ mice at 6-8 months was shorter than that of 2-4 months (*P* < 0.0001); further, the expression of p53 was higher, while the expression of PGC was lower [38]. The existence and effects of the “telomere-p53-PGC axis” have also been verified by other scholars [39, 40]. Accumulating evidence has shown that PGC-1*α* participate in the regulation of mitochondrial biogenesis and energy metabolism, which can directly reduce intracellular Ca^2+^ and indirectly reduce intracellular Ca^2+^ by inhibiting oxidative stress [12, 41, 42]. A recent study has argued that LTL is negatively associated with inflammation and oxidative status in humans [43]. In this study, we found that AF patients had a significantly lower level of PGC-1*α*mRNA expression and serum PGC-1*α* as well as decreased MMP when compared with controls, which indicate that telomere shortening and mitochondrial dysfunction are associated with the occurrence of aging-related AF. Interestingly, compared with the controls, the expression of leukocytes p53 mRNA was higher but without statistical difference; we assumed that this was because of the selected types of peripheral blood cells and the p53 expression being affected by many factors, as it is involved in multiple signaling pathways. Meanwhile, multivariate logistic regression confirmed that LTL and serum PGC-1*α* were inversely associated with the presence of AF, and linear regression analysis confirmed that LTL was significantly correlated with CRP and LAD. Therefore, we can reasonably speculate that telomere may regulate mitochondrial function, oxidative stress, calcium balance, and inflammation through downstream molecular PGC-1*α*, causing atrial electrical and structural remodeling, eventually inducing AF. The specific mechanism remains to be further studied.

AF is a global public-health challenge with increased mortality and major morbidity, including stroke and heart failure [1, 2]. With the aging of the population, an increasing segment is being directly or indirectly impacted by this common arrhythmia. Studies have shown that about 70% of AF occurs between 65 and 80 years of age. The incidence of AF increases with age, and males have a higher rate of it than females, which means that elderly males are at the highest risk for developing AF. Studies have confirmed that extensive asymptomatic AF screening in the elderly can reduce the incidence of stroke and related disabilities [44]. However, many elderly asymptomatic AF people cannot be diagnosed early due to a lack of effective biomarkers. Our study revealed that LTL and serum PGC-1*α* are negatively correlated with AF and confirmed that they have potential value in the early diagnosis of AF in the elderly male, which could provide new strategies for the screening of asymptomatic AF. In addition, PGC-1*α* is an important regulator of mitochondrial metabolism, which is a protective factor for AF in the elderly population; thus, PGC-1*α* may even be a possible novel target for AF intervention.

This study has several potential limitations. First, our study was an observational study, unable to establish a causal relationship. Second, many AF patients also presented with multiple age-related diseases such as hypertension, diabetes, and CHD; despite our best efforts to adjust for established and potential comorbidities, therefore, residual confounding by other unmeasured or unknown factors remains possible. Third, LTL was detected at one time point, while LTL declines throughout life, and the rate of telomere shortening could affect the incidence of AF. Fourth, due to various reasons, we were unable to detect the mitochondrial function indicators such as mitochondrial permeability transition pore (mPTP), mitochondrial calcium, and ROS for all subjects, and the specific mechanism remains to be further studied. Lastly, the subjects who we ultimately enrolled in our study were elderly males. More studies are warranted to compare these results with those of other populations.

In conclusion, LTL and serum PGC-1*α* are inversely correlated with the occurrence of aging-related AF, and mitochondrial dysfunction plays a role in this. These findings suggest that LTL and serum PGC-1*α* could possibly be novel predictive biomarkers for the occurrence or outcomes of aging-related AF.

## Figures and Tables

**Table 1:** Clinical and biochemical characteristics of AF patients and controls.

Characteristic	The controls (*n* = 96)	AF patients (*n* = 96)	*P* value
Clinical parameters			
Age (years)	77.81 ± 11.30	78.61 ± 11.64	0.629
Male, *n* (%)	96 (100)	96 (100)	1.000
BMI (kg/m^2^)	23.60 ± 2.83	24.25 ± 2.74	0.106
Smoking, *n* (%)	16 (16.67)	21 (21.88)	0.363
History of CHD, *n* (%)	21 (21.88)	18 (18.75)	0.593
Hypertension, *n* (%)	42 (43.75)	47 (48.96)	0.472
Diabetes mellitus, *n* (%)	15 (15.62)	17 (17.71)	0.700
Hyperlipidemia	24 (25.00)	20 (20.83)	0.495
Antihypertensive medication, *n* (%)	42 (43.75)	46 (47.92)	0.565
Glucose lowering treatment, *n* (%)	14 (14.58)	15 (15.63)	0.841
Lipid-lowering medication, *n* (%)	41 (42.71)	34 (35.42)	0.303
SBP (mmHg)	127.54 ± 11.10	129.02 ± 12.25	0.382
DBP (mmHg)	68.35 ± 8.74	70.42 ± 9.00	0.109
Laboratory parameters			
TC (mmol/L)	3.67 ± 0.73	3.59 ± 0.73	0.464
TG (mmol/L)	1.22 ± 0.49	1.34 ± 0.58	0.107
LDL-C (mmol/L)	2.33 ± 0.71	2.21 ± 0.68	0.236
HDL-C (mmol/L)	1.26 ± 0.33	1.21 ± 0.30	0.283
Cr (mmol/L)	84.03 ± 15.88	88.82 ± 21.12	0.077
BUN (mmol/L)	6.30 ± 1.56	6.55 ± 1.83	0.299
UA (mmol/L)	311.02 ± 58.10	333.08 ± 76.18	*0.025*
FBG (mmol/L)	6.18 ± 0.96	6.15 ± 0.99	0.829
GHb (%)	6.02 ± 0.57	6.11 ± 0.66	0.311
CRP (mg/dL)	0.71 ± 0.46	0.84 ± 0.51	0.077
Echocardiographic parameters			
LVEF (%)	60.06 ± 4.23	58.94 ± 3.87	0.056
LAD (mm)	37.54 ± 3.02	38.68 ± 4.02	*0.028*

Abbreviations: BMI: body mass index; CHD: coronary heart disease; SBP: systolic blood pressure; DBP: diastolic blood pressure; TC: total cholesterol; TG: triglyceride; LDL-C: low-density lipoprotein cholesterol; HDL-C: high-density lipoprotein cholesterol; Cr: creatinine; BUN: blood urea nitrogen; UA: uric acid; FBG: fasting blood glucose; GHb: glycated hemoglobin; CRP: C-reactive protein; LVEF: left ventricular ejection fraction; LAD: left atrial diameter. *P* < 0.05 with italic font means statistically significant.

**Table 2:** Clinical and biochemical characteristics of subjects without other diseases.

Characteristic	The controls (*n* = 26)	AF patients (*n* = 23)	*P* value
Clinical parameters			
Age (years)	77.54 ± 9.82	76.52 ± 12.11	0.530
Male, *n* (%)	26 (100)	23 (100)	1.000
BMI (kg/m^2^)	23.80 ± 2.75	24.12 ± 2.65	0.684
SBP (mmHg)	128.23 ± 10.58	124.96 ± 10.75	0.289
DBP (mmHg)	69.54 ± 8.12	69.13 ± 9.75	0.874
Laboratory parameters			
TC (mmol/L)	3.95 ± 0.61	3.71 ± 0.62	0.170
TG (mmol/L)	1.20 ± 0.54	1.40 ± 0.54	0.201
LDL-C (mmol/L)	2.42 ± 0.81	2.05 ± 0.61	0.078
HDL-C (mmol/L)	1.36 ± 0.33	1.20 ± 0.30	0.083
Cr (mmol/L)	79.27 ± 11.68	86.70 ± 22.32	0.145
BUN (mmol/L)	6.06 ± 1.08	6.61 ± 2.18	0.256
UA (mmol/L)	307.50 ± 49.88	301.61 ± 68.70	0.731
FBG (mmol/L)	6.03 ± 0.80	6.21 ± 1.13	0.518
GHb (%)	6.10 ± 0.69	6.10 ± 0.76	0.998
CRP(mg/dL)	0.65 ± 0.37	1.00 ± 0.56	*0.011*
Echocardiographic parameters			
LVEF (%)	61.31 ± 3.36	59.70 ± 3.32	0.098
LAD (mm)	37.35 ± 3.15	39.04 ± 3.39	0.076
Telomere-associated molecules			
LTL (*T*/*S* ratio)	3.23 ± 0.76	2.30 ± 0.73	*<0.001*
Leukocyte p53 mRNA	0.63 ± 0.28	0.69 ± 0.24	0.395
Leukocyte PGC-1*α* mRNA	4.35 ± 1.00	3.52 ± 0.89	*0.004*
Serum PGC-1*α* (pg/mL)	488.69 ± 65.94	420.56 ± 84.79	*0.003*

Abbreviations: BMI: body mass index; SBP: systolic blood pressure; DBP: diastolic blood pressure; TC: total cholesterol; TG: triglyceride; LDL-C: low-density lipoprotein cholesterol; HDL-C: high-density lipoprotein cholesterol; Cr: creatinine; BUN: blood urea nitrogen; UA: uric acid; FBG: fasting blood glucose; GHb: glycated hemoglobin; CRP: C-reactive protein; LVEF: left ventricular ejection fraction; LAD: left atrial diameter. *P* < 0.05 with italic font means statistically significant.

**Table 3:** Logistic regression analysis for the presence of AF.

	Simple regression		Multiple regression	
	OR (95% CI)	*P* value	OR (95% CI)	*P* value
Clinical parameters				
Age (years)	1.006 (0.982-1.031)	0.627		
BMI (kg/m^2^)	1.089 (0.982-1.207)	0.107		
Smoking, *n* (%)	0.714 (0.347-1.471)	0.361		
History of CHD, *n* (%)	0.824 (0.407-1.668)	0.591		
Hypertension, *n* (%)	1.233 (0.699-2.177)	0.470		
Diabetes mellitus, *n* (%)	1.162 (0.543-2.486)	0.699		
Hyperlipidemia	0.789 (0.402-1.551)	0.493		
Antihypertensive medication, *n* (%)	1.183 (0.670-2.088)	0.562		
Glucose lowering treatment, *n* (%)	1.085 (0.492-2.391)	0.840		
Lipid-lowering medication, *n* (%)	0.736 (0.411-1.316)	0.301		
SBP (mmHg)	1.011 (1.008-1.061)	0.380		
DBP (mmHg)	1.027 (0.994-1.061)	0.110		
Laboratory parameters				
TC (mmol/L)	0.864 (0.584-1.277)	0.462		
TG (mmol/L)	1.562 (0.904-2.700)	0.110		
LDL-C (mmol/L)	0.780 (0.517-1.176)	0.236		
HDL-C (mmol/L)	0.607 (0.244-1.509)	0.282		
Cr (mmol/L)	1.015 (0.998-1.031)	0.084		
BUN (mmol/L)	1.095 (0.923-1.298)	0.300		
UA (mmol/L)	1.005 (1.001-1.009)	*0.027*	1.004 (0.999-1.009)	0.130
FBG (mmol/L)	0.968 (0.723-1.296)	0.828		
GHb (%)	1.272 (0.798-2.026)	0.311		
CRP (mg/dL)	2.250 (1.277-3.965)	*0.005*	1.971 (1.023-3.799)	*0.043*
Echocardiographic parameters				
LVEF (%)	0.933 (0.869-1.002)	0.058		
LAD (mm)	1.095 (1.009-1.187)	*0.030*	1.084 (0.984-1.195)	0.103
Telomere-associated molecules				
LTL (*T*/*S* ratio)	0.388 (0.269-0.560)	<*0.001*	0.404 (0.278-0.587)	<*0.001*
Leukocyte p53 mRNA	2.340 (0.848-6.457)	0.101		
Leukocyte PGC-1*α* mRNA	2.340 (0.848-6.457)	0.101		
Serum PGC-1*α*(pg/mL)	0.994 (0.991-0.998)	*0.001*	0.994 (0.989-0.998)	*0.003*

Abbreviations: CI: confidence interval; BMI: body mass index; CHD: coronary heart disease; SBP: systolic blood pressure; DBP: diastolic blood pressure; TC: total cholesterol; TG: triglyceride; LDL-C: low-density lipoprotein cholesterol; HDL-C: high-density lipoprotein cholesterol; Cr: creatinine; BUN: blood urea nitrogen; UA: uric acid; FBG: fasting blood glucose; GHb: glycated hemoglobin; CRP: C-reactive protein; LVEF: left ventricular ejection fraction; LAD: left atrial diameter; *T*/*S* ratio: the ratio of telomere repeats to single-copy gene copies; PGC-1*α*: peroxisome proliferator-activated receptor *γ* coactivator-1*α*. *P* < 0.05 with italic font means statistically significant.

**Table 4:** Logistic regression analysis in subgroup.

	Simple regression		Multiple regression	
	OR (95% CI)	*P* value	OR (95% CI)	*P* value
Age (years)	1.017 (0.965-1.072)	0.521	0.996 (0.917-1.081)	0.920
CRP(mg/dL)	6.021 (1.319-27.485)	*0.020*	9.163 (0.772-108.764)	0.790
LTL (*T*/*S* ratio)	0.162 (0.054-0.488)	*0.001*	0.188 (0.046-0.766)	*0.020*
Leukocyte PGC-1*α* mRNA	0.405 (0.208-0.788)	*0.008*	0.460 (0.196-1.080)	0.075
Serum PGC-1*α*(pg/mL)	0.987 (0.978-0.997)	*0.008*	0.983 (0.968-0.998)	*0.031*

Abbreviations: CI:, confidence interval; CRP: C-reactive protein; *T*/*S* ratio: the ratio of telomere repeats to single-copy gene copies; PGC-1*α*: peroxisome proliferator-activated receptor *γ* coactivator-1*α*. *P* < 0.05 with italic font means statistically significant.

**Table 5:** The correlation of LTL with other clinical characteristics.

	Simple linear regression		Multiple linear regression	
	*r*	*P*	*β*	*P*
Clinical parameters				
Age (years)	-0.151	*0.037*	-0.111	0.110
BMI (kg/m^2^)	-0.079	0.274		
Smoking, *n* (%)	0.119	0.099		
History of CHD, *n* (%)	-0.013	0.861		
Hypertension, *n* (%)	-0.046	0.529		
Diabetes mellitus, *n* (%)	0.115	0.114		
Hyperlipidemia	0.128	0.077		
Antihypertensive medication, *n* (%)	-0.045	0.532		
Glucose lowering treatment, *n* (%)	0.103	0.155		
Lipid-lowering medication, *n* (%)	-0.025	0.726		
SBP (mmHg)	0.068	0.352		
DBP (mmHg)	-0.071	0.331		
Laboratory parameters				
TC (mmol/L)	0.015	0.836		
TG (mmol/L)	-0.046	0.523		
LDL-C (mmol/L)	0.041	0.571		
HDL-C (mmol/L)	0.097	0.180		
Cr (mmol/L)	0.020	0.785		
BUN (mmol/L)	0.040	0.577		
UA (mmol/L)	-0.138	0.057		
FBG (mmol/L)	0.071	0.329		
GHb (%)	0.047	0.519		
CRP (mg/dL)	-0.200	*0.005*	-0.167	*0.017*
Echocardiographic parameters				
LVEF (%)	0.021	0.771		
LAD (mm)	-0.196	*0.006*	-0.165	*0.018*
Telomere-associated molecules				
Leukocyte p53 mRNA	0.059	0.417		
Leukocyte PGC-1*α* mRNA	0.168	*0.020*	0.131	0.160
Serum PGC-1*α* (pg/mL)	0.176	*0.014*	0.109	0.122

Abbreviations: BMI: body mass index; CHD: coronary heart disease; SBP: systolic blood pressure; DBP: diastolic blood pressure; TC: total cholesterol; TG: triglyceride; LDL-C: low-density lipoprotein cholesterol; HDL-C: high-density lipoprotein cholesterol; Cr: creatinine; BUN: blood urea nitrogen; UA: uric acid; FBG: fasting blood glucose; GHb: glycated hemoglobin; CRP: C-reactive protein; LVEF: left ventricular ejection fraction; LAD: left atrial diameter; PGC-1*α*: peroxisome proliferator-activated receptor *γ* coactivator-1*α*. *P* < 0.05 with italic font means statistically significant.

## Data Availability

The data used to support the findings of this study are available from the corresponding authors upon request.
